# Regulation of Septin Dynamics by the *Saccharomyces cerevisiae* Lysine Acetyltransferase NuA4

**DOI:** 10.1371/journal.pone.0025336

**Published:** 2011-10-03

**Authors:** Leslie Mitchell, Andrea Lau, Jean-Philippe Lambert, Hu Zhou, Ying Fong, Jean-François Couture, Daniel Figeys, Kristin Baetz

**Affiliations:** Ottawa Institute of Systems Biology, Department of Biochemistry, Microbiology, and Immunology, University of Ottawa, Ottawa, Ontario, Canada; University of Edinburgh, United Kingdom

## Abstract

In the budding yeast *Saccharomyces cerevisiae*, the lysine acetyltransferase NuA4 has been linked to a host of cellular processes through the acetylation of histone and non-histone targets. To discover proteins regulated by NuA4-dependent acetylation, we performed genome-wide synthetic dosage lethal screens to identify genes whose overexpression is toxic to non-essential NuA4 deletion mutants. The resulting genetic network identified a novel link between NuA4 and septin proteins, a group of highly conserved GTP-binding proteins that function in cytokinesis. We show that acetyltransferase-deficient NuA4 mutants have defects in septin collar formation resulting in the development of elongated buds through the Swe1-dependent morphogenesis checkpoint. We have discovered multiple sites of acetylation on four of the five yeast mitotic septins, Cdc3, Cdc10, Cdc12 and Shs1, and determined that NuA4 can acetylate three of the four *in vitro*. *In vivo* we find that acetylation levels of both Shs1 and Cdc10 are reduced in a catalytically inactive *esa1* mutant. Finally, we determine that cells expressing a Shs1 protein with decreased acetylation *in vivo* have defects in septin localization that are similar to those observed in NuA4 mutants. These findings provide the first evidence that yeast septin proteins are acetylated and that NuA4 impacts septin dynamics.

## Introduction

Lysine acetylation is traditionally associated with histone proteins and the regulation of a variety of chromatin-based cellular processes [1]. More recently, systematic efforts have revealed that thousands of additional proteins are acetylated *in vivo*, dramatically altering our view of the abundance of this modification and the multitude of cellular pathways it impacts [2], [3], [4], [5]. The next major challenge will be to connect lysine acetyltransferase (KAT) enzymes to their substrates and target lysine residues in order to understand the biological consequences of acetylation.

NuA4 is a KAT complex in the budding yeast *S. cerevisiae* [6] whose function has been linked to a variety of cellular processes including transcription [7], DNA repair [8], chromosome segregation [9], cell cycle control [10], [11], stress response [12], [13], vesicle-mediated transport [13] and gluconeogenesis [14]. Accordingly, the orthologous human complex, Tip60, which is highly conserved both structurally and functionally [15], is known to be misregulated in a number of human pathologies, including Alzheimer's disease [16] and cancer [17]. NuA4 is a multi-subunit complex, composed of the essential catalytic subunit Esa1 [6], five other essential subunits Act1, Arp4, Epl1, Swc4, Tra1, and seven non-essential subunits Eaf1, Eaf3, Eaf5, Eaf6, Eaf7, Yaf9, and Yng2. Molecular dissection has revealed NuA4 to be modular in nature, and that assembly of its multiple sub-complexes depends on the Eaf1 subunit [18]; in the absence of Eaf1, NuA4 complex integrity is disrupted [13]. The Eaf3/Eaf5/Eaf7 and Act1/Arp4/Swc4/Yaf9 sub-complexes, along with the final subunit, Tra1, have been proposed to function as a recruitment module that targets the acetyltransferase activity of the complex, contained in the final sub-complex (Esa1/Epl1/Yng2/Eaf6), also known as Piccolo NuA4 (PicNuA4), to specific loci [7]. Moreover, PicNuA4 exists as a discrete, acetyltransferase-competent complex in wild type yeast cells independent of the larger NuA4 complex, and is thought to globally and non-specifically acetylate histones [19]. Importantly, the non-essential proteins Eaf1 and Yng2 are each required for Esa1's acetyltransferase activity on histone targets *in vivo* [9], [20], [21] and can therefore serve as genetic tools for studying NuA4-related functions.

*In vivo* the most thoroughly characterized acetylation targets of Esa1 include the histone proteins H4 [6], [22], [23], and the H2A variant Htz1 [20], [21], [24]. Notably, Esa1 must be associated with PicNuA4 or the full NuA4 complex to be catalytically active on nucleosome-bound histones [19], [22]. While a plausible explanation to account for the many and diverse cellular functions of NuA4 is that the complex acts as a master transcriptional regulator in the cell via histone acetylation alone, microarray analysis suggests the role of NuA4 in global gene expression is relatively minor [9], [12], [25]. One notable exception is the positive contribution NuA4 makes in rDNA gene transcription via histone H4 acetylation at ribosomal promoters [26]. An alternative hypothesis, which is strongly supported by multiple lines of evidence, is that NuA4 mediates many of its diverse cellular effects through the acetylation of non-histone targets. To date, two non-histone NuA4 acetylation targets have been characterized *in vivo*: the NuA4 and PicNuA4 subunit Yng2 [27], and the gluconeogenesis regulator Pck1 [14]. In these two cases, Esa1-dependent acetylation regulates protein stability and enzymatic activity, respectively. Similarly, several non-histone acetylation targets have been documented *in vivo* for the human NuA4 complex, Tip60, including p53, c-myc, and ATM kinase [28], [29]. Finally, to explore the possibility that NuA4 acetylates a broad range of both nuclear and non-nuclear proteins, Lin *et al.* [14], using a protein acetylation microarray, identified 91 novel *in vitro* substrates of NuA4 and further confirmed 13 non-chromatin proteins to be substrates of NuA4 *in vivo*. However, the full extent and the biological consequences of NuA4 dependent *in vivo* acetylation have yet to be established.

To date, NuA4 function has been assessed extensively using genome-wide synthetic lethal (SL) genetic interaction screens [13], [27], [30], [31], [32], [33], which highlight genetic redundancy between pairs of hypomorphic gene mutations. Though highly informative in regards to biological function, SL screens generally identify proteins that function in parallel rather than direct pathways [34]. Here we have taken advantage of a new genome-wide genetic screen called synthetic dosage lethal (SDL) profiling, which systematically assesses the effect of gene overexpression in a mutant background [35]. It is now well established that SDL and SL genetic interactions probe mechanistically unique genetic relationships [35], [36], [37], indicating SDL profiling can be used as a complementary tool to predict novel processes impacted by query genes of interest. Further, based on the premise that an increased amount of inappropriately modified substrate may cause a measurable fitness defect specifically in an enzyme mutant background, genome-wide SDL screens have proven successful in identifying downstream targets of kinases [35], [37].

Thus, in an attempt to discover novel pathways and putative substrates directly regulated by NuA4, we have generated SDL profiles for the seven non-essential NuA4 deletion mutants. Our network uncovers a novel connection between NuA4 and septin proteins, a group of highly conserved proteins that function in cytokinesis in many organisms [38]. We discover that acetylation-deficient *eaf1Δ* cells develop elongated buds and have defects in septin dynamics. Further, we identify multiple acetyl lysine residues on four of the five mitotic septin proteins, that NuA4 can acetylate three septin proteins *in vitro*, and that the acetylation level of at least two septin proteins is dependent on Esa1 *in vivo*. Finally, we focus on the biological consequence of Shs1 acetylation and determine that cells expressing an unacetylable Shs1-HA_3_ fusion protein displays bud morphology and septin localization defects similar to those observed in NuA4 mutants suggesting a direct relationship between septin acetylation and septin dynamics. Importantly, this work establishes a new role for NuA4 in the regulation of septin dynamics and identifies acetylation as a novel post-translational modification regulating septin proteins.

## Materials and Methods

### Yeast strains and plasmids

Yeast strains used in this study are described in Table S1 and plasmids generated for this study listed in Table S2. The galactose-inducible overexpression array [35] was a gift from Brenda Andrews. Genomic deletion or epitope tag integrations made for this study were designed with PCR-amplified cassettes as previously described and confirmed by PCR analysis [39].

### SDL screening

Media used for the genome-wide SDL screens was prepared as described [35], except that as the five non-essential NuA4 deletion mutants used for genome-wide SDL analysis were linked to the dominant selectable marker NAT, diploid and final haploid selections were achieved using clonNAT (Werner Bioagents, 5.0000) at a final concentration of 100ug/mL. The SDL library (∼5300 yeast strains) was initially arrayed in duplicate at a density of 1536 colonies per plate, yielding 9 high-density library plates maintained on medium lacking uracil to select for the presence of the overexpression plasmids. All replica pinning steps were carried out as described [35] using a Singer RoToR HDA (Singer Instrucments). Genome-wide SDL screens were performed in triplicate at 25°C using the following query strains: *eaf1Δ* (YKB622), *eaf3Δ* (YKB995), *eaf5Δ* (YKB852), *eaf6Δ* (YKB623), *eaf7Δ* (YKB853). For the final scoring analysis, plate images were acquired using the ChemiDoc XRS Molecular Imaging System (BioRad) two days after pinning onto synthetic medium lacking uracil and containing either glucose or galactose as the sugar source. Images were analyzed using an in-house automated scoring method. SDL interactions that appeared in a list of “toxic genes”, whose overexpression alone is known to kill wild type cells [35], were discarded from further analysis. For each query strain, interactions identified in at least two screens were confirmed by direct transformation followed by serial spot dilution assays. All SDL and synthetic dosage sick (SDS) interactions were then directly tested in all seven non-essential NuA4 deletion mutant backgrounds, *eaf1Δ* (YKB44), *eaf3Δ* (YKB1162), *eaf5Δ* (YKB658), *eaf6Δ* (YKB504), *eaf7Δ* (YKB530), *yaf9Δ* (KYB464) and *yng2Δ* (YKB494), by direct transformation followed by streak test.

### Serial spot dilution assay to confirm SDL interactions

Wild type and NuA4 mutant strains, transformed with galactose-inducible overexpression plasmids or an empty vector control (pRS416) using traditional methods [40], were grown to mid-log phase in SD-URA liquid medium at 25°C. Ten-fold serial dilutions (OD_600_ = 0.1, 0.01, 0.001, 0.0001) were spotted onto medium lacking uracil and containing either galactose or glucose. Plates were incubated for 5 days at 25°C and images were collected using the ChemiDoc XRS Molecular Imaging system (Biorad).

### Microscopy

All cells were grown to mid-log phase in YPD supplemented with adenine unless otherwise noted. For imaging of fixed cells, samples were prepared with paraformaldehyde as previously described [41] and Z-stack images were collected (0.3 µm steps) across 5–6 µm. For *in vivo* time-lapse studies, cells were re-suspended at high density in SD media and then spread on agarose pads prepared with synthetic complete media on standard microscope slides prepared as described [42], except coverslips were sealed with VALAP (1 vasoline: 1 lanolin: 1 paraffin). Live cell imaging was performed at room temperature and Z-stacks (0.5 µm steps) were collected across 20 µm every 5 minutes for wild type cells up to 2 hours and every 15 minutes for *eaf1Δ* cells up to 6 hours. For quantification, only the mother cells were tracked post cytokinesis. Bud morphology and septin localization was scored for 15 wild type cells from a single experiment (1 field of view) and from 4 unique experiments for *eaf1Δ* cells (between 4 and 15 cycling cells were scored in each of 4 fields of view to collect data for 43 cells). Microscopy was performed using a Leica DMI 6000 florescent microscope (Leica Microsystems GmbH, Wetzler Germany), equipped with a Sutter DG4 light source (Sutter Instruments, California, USA), Ludl emission filter wheel with Chroma band pass emission filters (Ludl Electronic Products Ltd., NY, USA) and Hamamatsu Orca AG camera (Hamamatsu Photonics, Herrsching am Ammersee, Germany). Images were collected and analyzed using Velocity 4.3.2 Build 23 (Perkin Elmer). Analysis was performed on images collapsed into two dimensions using the “extended focus” option in Velocity.

### GFP immunopurification and *in vivo* acetylation

All cells were grown to mid-log phase in YPD supplemented with adenine unless otherwise noted in 50–100 mL cultures. Lysate preparation and immunopurifications were carried out as described for septin TAP immunopurifications, except lysates were sonicated 1×10 sec and 20 µL of GFP-trap magnetic beads (Chromotek, GTM-20) was added to 10 mg of whole cell lysate. Proteins were eluted in 25 µl of 2x loading dye (50 mM Tris, pH 6.8, 2% SDS, 0.1% bromophenol blue, 200 mM beta-2-mercaptoethanol, 10% glycerol) with heating at 65°C for 10 minutes. Western blotting was performed using a semi-dry transfer apparatus from Biorad (Trans-Blot SD Semi Dry Electrophoretic Transfer Cell; 170-3940) and buffers recommended by the manufacturer. Protein transfer was performed for 1 hour using constant milliamps (mA) based on the following calculation: 0.8mA x area of gel x # of gels. Acetylation was detected using two anti-acetyl lysine antibodies at the following dilutions: Cell Signaling, 1/500 (9681); Upstate 1/1000 (06-933). Other primary antibodies used were: α-Act1 1/1000 (Novus Biologicals, NB600-505); α-MYC 1/1000 (Roche, 11667149001); α-G6PDH 1/10000 (Sigma, A9521), α-HA 1/1000 (Covance, MMS101P); α-GFP 1/1000 (Roche, 11814460001); 1/1000 α-hyperacetylated histone H4 (06-946). All blocking, primary, and secondary antibody incubations were performed with 5% milk dissolved in PBS plus 0.1% Tween20 (PBS-T), except the Cell Signaling anti-acetyl lysine antibody, in which the manufacturer's recommendation of 5% BSA in TBS plus 0.1% Tween20 was followed. Primary incubations were carried out overnight at 4°C and secondary incubations at room temperature for 2 hours. Secondary antibodies, all used at a dilution of 1/5000, were peroxidase-conjugated goat anti-rabbit IgG (Chemicon; AP307P), and peroxidase-conjugated goat anti-mouse IgG (Bio-Rad; 170-6516) where appropriate. Chemiluminescence was detected using Immobilon Western Chemiluminescent HRP substrate (Millipore; WBKLS0500) and developed on a ChemiDoc XRS system (Biorad; 170-8070). All experiments were repeated a minimum of three times and a representative image is shown.

### Septin TAP Immunopurification and radioactive *in vitro* KAT assay

Septin protein complexes were isolated from exponentially growing yeast cultures via single step affinity purification of protein A, one epitope of the tandem affinity purification (TAP) tag. Cells from 300 ml of mid-log phase culture grown in YPD at 30°C were collected by centrifugation, washed in 25 mL of ice-cold water and transferred to 1.5 mL Eppendorf tubes where they were flash frozen on dry ice. Cell pellets were re-suspended in 300 µL of lysis buffer (100 mM HEPES pH 8.0, 20 mM magnesium acetate, 10% glycerol (V/V), 10 mM EGTA, 0.1 mM EDTA, 300 mM sodium acetate, and fresh protease inhibitor cocktail (Sigma, P8215)) plus an equal volume of glass beads, and cells were lysed through vortexing (six 1-minute blasts with incubation on ice between vortexing). Lysates were subjected to sonication (3×20 sec; 1 minute incubation on ice between each pulse) using a Misonix Sonicator 3000 at setting four. Prior to centrifugation (10 min, 3000 rpm, 4°C), Nonidet P-40 was added to a final concentration of 1%. Forty milligrams of whole cell extract was incubated with 100 µL of magnetic beads (Invitrogen, 143.02D) cross-linked to rabbit immunoglobulin (IgG) (Chemicon, PP64) as per the manufacturer's instructions. Following 2 hours of end-over-end rotation at 4°C, the beads were collected on a magnet and washed 3 times with 1 mL of ice cold wash buffer (100 mM HEPES pH 8.0, 20 mM magnesium acetate, 10% glycerol (V/V), 10 mM EGTA, 0.1 mM EDTA, 300 mM sodium acetate, 0.5% Nonidet P-40). The KAT assay was performed with the septins proteins bound to the magnetic beads. To the septin-magnetic bead matrix was added 3 µL of 5X KAT buffer (250 mM Tris pH 8.0, 250 mM NaCl, 25 mM MgCl_2_, 5 mM DTT), 0.5 µCi of [^3^H]-acetyl coenzyme A (Perkin-Elmer, NET290L050UC), and 5 µL of NuA4, purified from yeast cells (described below) in a final volume of 15 µL. The reaction was incubated for 1 hour at 30°C with end-over-end rotation. As the NuA4 preparation included the TEV enzyme, TAP-tagged septin proteins were cleaved from the magnetic beads during the KAT assay. An equal volume of 2x loading dye (100 mM Tris, pH 6.8, 4% SDS, 0.2% bromophenol blue, 200 mM β-2-mercaptoethanol, 20% glycerol) was added and the samples heated at 65°C for 10 minutes. Proteins were separated by SDS-PAGE (7.5%). Following Coomassie staining and destaining, an image was taken of the gel. Next, the gel was treated for fluorography, dried, and exposed to film for 1 month at −80°C. To ensure NuA4′s activity, a control reaction was performed on 2 µg of chicken core histones (Upstate, 13-107) using 3 µL of purified NuA4 and 5 µCi of [^3^H]-acetyl coenzyme A in a final volume of 10 µL. This sample was processed as described above except proteins were separated using 15% SDS-PAGE.

### Purification of NuA4 from yeast for *in vitro* KAT assays

Endogenously TAP-tagged Esa1 was used to purify NuA4 from yeast cells as described [13], except the immunopurified products were eluted from magnetic beads by enzymatic cleavage in TC Buffer (50 mM Tris, pH 8.0, 1 mM DTT, 0.1% Nonidet P-40, 150 mM NaCl, 10% glycerol) using tobacco etch virus (TEV; prepared in-house). Briefly, cells from 1 L of exponentially growing culture (in YPD, at 30°C) were lysed and NuA4 was purified in a single step using magnetic beads coupled to IgG. After washing, the NuA4-bead matrix was resuspended in TC buffer (100 µL) to which was added 20 µL of TEV. The cleavage reaction was incubated overnight at 4°C with end-over-end rotation. Finally, the supernatant was isolated from the beads, aliquoted, and stored at −80°C. The purity of the NuA4 preparation was assessed by silver stain using 2 µL of TEV-cleaved sample separated by SDS-PAGE (7.5%) and its acetyltransferase activity was tested in an *in vitro* KAT assay (described above) using 2 µg of chicken core histones (Upstate, 13-107) and 2 µg of acetyl coenzyme A (Sigma, A2056) in a final volume of 15 µL. The acetylation signal was assessed by Western blot using an anti-acetyl lysine antibody (Upstate, 06-933).

### Detection of acetylated lysine residues by mass spectrometry

TAP-tagged septin proteins were immunopurified as described above for the radioactive KATs assays. One half of each sample was subjected to an *in vitro* NuA4 KAT assay, as described above, except 1 µg of unlabelled acetyl coenzyme A (Sigma, A2056) was added. Samples were resolved on SDS-PAGE gel, silver stained and bands excised. Following in-gel digestion with trypsin, samples were analyzed by LC-MS/MS using an Agilent 1100 HPLC system (Agilent Technologies) coupled to either an LTQ or an LTQ-Orbitrap XL spectrometer (Thermo-Electron) as indicated (Table S5). Acetylated lysine residues on septin proteins were identified as previously described [4]. MS/MS corresponding to putative lysine acetylation sites were all manually validated.

### Cloning and mutagenesis of *SHS1*

*SHS1* was C-terminally epitope tagged (HA_3_) at its endogenous genomic locus using standard homologous recombination techniques [39]. The *SHS1-HA_3_* gene fusion plus endogenous promoter were subsequently amplified from isolated genomic DNA using Phusion Polymerase (Finnzyme, F-530S) with a forward primers localized ∼500 base pair upstream of the *SHS1-HA_3_* start site and a reverse primer that bound 3′ of the transcriptional terminator sequence of the *HA_3_* coding sequence. Each primer contained a 5′ *Sal*I recognition sequence, thus the resulting PCR product was digested with *Sal*I, gel purified (Qiagen, 28704), and ligated into the pRS415 vector [43] that was previously digested with *Sal*I and treated with calf intestinal phosphatase (NEB, M0290S). Ligation reactions were carried out overnight with T4 DNA ligase at 16°C (NEB, M0202S), and then transformed into competent *E.coli* DH5alpha cells prepared in-house. The final clone was fully sequenced. HA_3_ and C-terminal truncation mutants were generated as described [44]. Point mutations were generated using the QuikChange Multi Site-Directed Mutagenesis Kit (Agilent, 200514). The successful introduction of all point mutations was confirmed by sequencing.

## Results

### The NuA4 synthetic dosage lethal network

**Figure 1:**
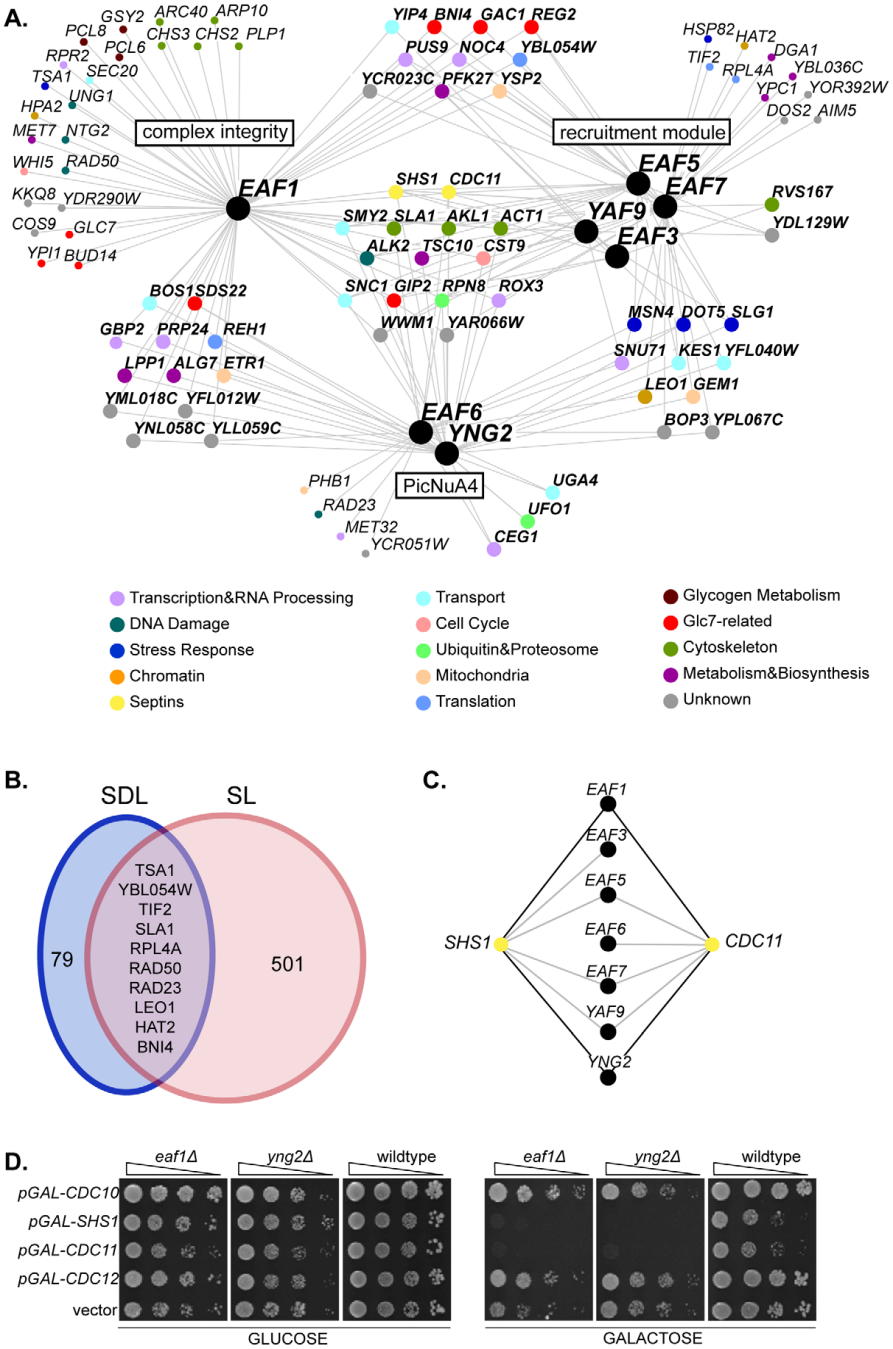
The NuA4 synthetic dosage lethal interaction network identifies a novel link with septin proteins. (**A**) The NuA4 SDL genetic interaction network. Black nodes represent NuA4 genes, which are organized based on sub-complexes within the NuA4 complex (*EAF1*: complex integrity; *EAF3, EAF5, EAF7, YAF9*: recruitment module; *EAF6, YNG2*: Piccolo NuA4, PicNuA4). Interacting genes are represented by nodes colour-coded according to functional annotation as listed in the legend, and organized into groups based on the number of interactions with NuA4 sub-complexes. For instance, nodes located in the centre of the figure interact with one or more NuA4 mutants from each of the three sub-complexes. Small nodes denote genes that interact with only one NuA4 mutant. Edges indicate SDL or SDS genetic interactions (see also Table S3). (**B**) Comparison of the NuA4 SDL and SL genetic interactions identified by genome-wide screens for the five non-essential NuA4 subunits *EAF1*, *EAF3*, *EAF5*, *EAF6*, *EAF7* (see Table S4 for the compiled list of SL interactions). (**C**) Overexpression of the septin genes *CDC11* and *SHS1* (yellow nodes) is toxic to most non-essential NuA4 deletion mutants (black nodes). Black and grey edges represent SDL or SDS interactions, respectively. (**D**) Overexpression of the septin genes *CDC11* and *SHS1* cause death in the absence of *EAF1* or *YNG2*. Isogenic wild type (YKB779), *eaf1Δ* (YKB44) and *yng2Δ* (YKB494) cells containing galactose-inducible, plasmid-borne copy of galactose inducible *CDC10*, *SHS1*, *CDC11*, *CDC12*, or an empty vector control (pRS416) were spotted in serial ten-fold dilutions on minimal media lacking uracil and containing either glucose or galactose at 25°C for 3 or 5 days, respectively.

To elucidate pathways regulated by the NuA4 lysine acetyltransferase complex and to discover putative substrates, we identified SDL interactions for the seven non-essential NuA4 subunits. Genome-wide SDL analysis was performed with five NuA4 query genes (*eaf1Δ*, *eaf3Δ*, *eaf5Δ*, *eaf6Δ*, *eaf7Δ*) using synthetic genetic array (SGA) technology [35], [45]. Any gene whose overexpression caused an SDL phenotype in at least one NuA4 query strain was directly tested in all non-essential NuA4 deletion mutant backgrounds, including the remaining two, *yaf9Δ* and *yng2Δ*. The resulting NuA4 SDL genetic network encompasses 173 interactions among 89 genes, of which 16% (27/173) are SDL interactions and the remainder are SDS (synthetic dosage sick) interactions. (Figure 1A, Table S3). Similar to previous NuA4 SL screens [13], [27], [30], [31], our SDL analysis successfully identified biologically relevant genes in cellular processes known to be impacted by NuA4, such as transcription [7], DNA damage [8], cell cycle progression [10], [11], and the stress response [12], [13]. Given that the genetic underpinnings of SDL and SL interactions are unique [35], [37], as expected there was only minimal overlap between the NuA4 SDL interactions with previously published genome-wide SL interactions (Figure 1B and Table S4) [13], [27], [30], [32], [33].

As most non-essential NuA4 subunits participate in multiple protein complexes in yeast [7], we were particularly interested in genes that displayed SDL interactions with multiple NuA4 deletion mutants. We grouped interacting genes with respect to the three major functional sub-complexes within NuA4: Eaf1, the subunit required for complex assembly [13], [18]; Yng2 and Eaf6 as subunits of PicNuA4 [13], [19]; and the remaining non-essential subunits as components of the recruitment domain [7] (Figure 1A). While more than half of genes in the network interacted with mutants from at least two sub-complexes (47/89), 15 genes displayed SDL interactions with at least one NuA4 mutant from each of the sub-complexes (Figure 1A). We focused our attention on two genes within the set of 15 highly connected interactions, *CDC11* and *SHS1,* for two reasons. First, overexpression of each of *CDC11* and *SHS1*, which cause moderately impaired growth in wild type cells as previously reported [35], [46], was toxic to six of the seven non-essential NuA4 deletion mutants (Figure 1C). Only one other gene in the SDL network, *RPN8*, displayed this same degree of SDL interconnectivity with NuA4. Secondly, *CDC11* and *SHS1* encode proteins that function within a single complex; along with *CDC3*, *CDC10* and *CDC12*, these genes encode the five mitotic septin proteins in yeast. Septins are highly conserved GTP-binding proteins that form hetero-octameric protein complexes localized to the bud neck in yeast [38], [47], [48]. Notably, overexpression of *CDC11* and *SHS1* was lethal to the NuA4 acetyltransferase-deficient mutant cells *eaf1Δ* and *yng2Δ* (Figure 1D), suggesting the acetyltransferase activity associated with the full NuA4 complex is required to buffer the effects of *SHS1* and *CDC11* overexpression. However, the toxic effects of septin gene overexpression are specific, as neither *CDC10* nor *CDC12* overexpression resulted in a SDL phenotype with any NuA4 non-essential deletion mutant (Figure 1D and data not shown). The numerous SDL interactions between non-essential NuA4 mutants and *CDC11* and *SHS1* suggest a role for NuA4 in the regulation of septin protein function.

### The elongated bud morphology of *eaf1Δ* cells is dependent on Swe1

**Figure 2:**
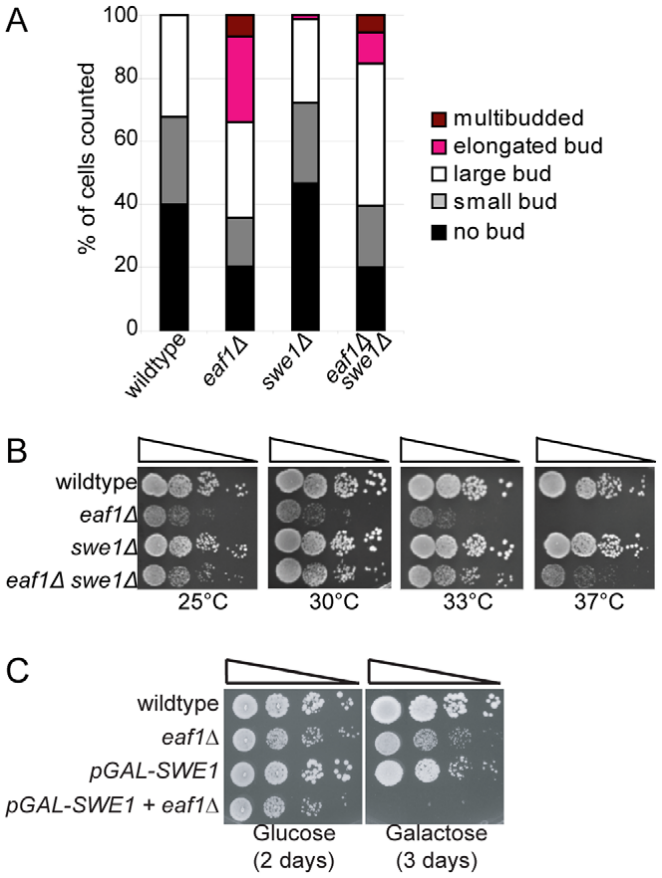
Elongated buds and temperature sensitivity of *eaf1Δ* cells is partially suppressed by deletion of *SWE1*. (**A**) *eaf1Δ* mutants exhibit elongated buds that can be suppressed by deletion of the Swe1 morphogenesis checkpoint. Wild type (YKB779), *eaf1Δ* (YKB42), *swe1Δ* (YKB1807), and *eaf1Δ swe1Δ* (YKB1266) cells were grown to mid-log phase in YPD at 25°C, fixed, and bud morphology was scored for at least 100 cells of each type. The average of three replicates is presented. (**B**) Deletion of *SWE1* rescues the temperature sensitivity of *eaf1Δ* cells. The strains used in (A) were spotted in ten-fold serial dilutions and incubated at the temperatures indicated on YPD plates. (**C**) Overexpression of *SWE1* is toxic to *eaf1Δ* cells. Wild type (YKB779), *eaf1Δ* (YKB42), *pGAL-SWE1* (YKB1648), and *eaf1Δ pGAL-SWE1* (YKB1806) cells were spotted on complete medium (yeast extract, peptone) in 10-fold serial dilutions containing glucose or galactose as the sugar source at 25°C for 2 days.

A hallmark phenotype associated with mutations in septin genes and their regulators is the development of elongated buds [49], [50], which results from activation of the Swe1-dependent morphogenesis checkpoint [51]. Briefly, this checkpoint coordinates mitotic progression and bud growth through the Swe1 kinase, which inhibits mitosis via phosphorylation and inactivation of the mitotic Cdk1 (Cdc28). As a consequence, cells accumulate in G2/M with hyper-elongated buds due to an inability to switch from apical to isotropic growth. We reasoned that if NuA4 regulates septins, then NuA4 mutants might display Swe1-dependent bud morphology defects. To test this, we assessed bud morphology of wild type and *eaf1Δ* cells. We discovered that ∼25% of *EAF1* deletion mutants displayed elongated buds as compared to wild type cells and that inactivation of the morphogenesis checkpoint via deletion of *SWE1* partially suppressed both the elongated bud morphology (Figure 2A) and temperature sensitivity of *eaf1Δ* cells (Figure 2B). Conversely, constitutive activation of the checkpoint induced by overexpression of *SWE1* was lethal to *eaf1Δ* cells (Figure 2C). This data suggests that one of the defects associated with *eaf1Δ* cells is activation of the morphogenesis checkpoint. As these phenotypes are shared with septin mutants and their regulators, this supports a role for NuA4 in septin regulation.

### Acetyltransferase-deficient NuA4 mutants have defects in septin dynamics

**Figure 3:**
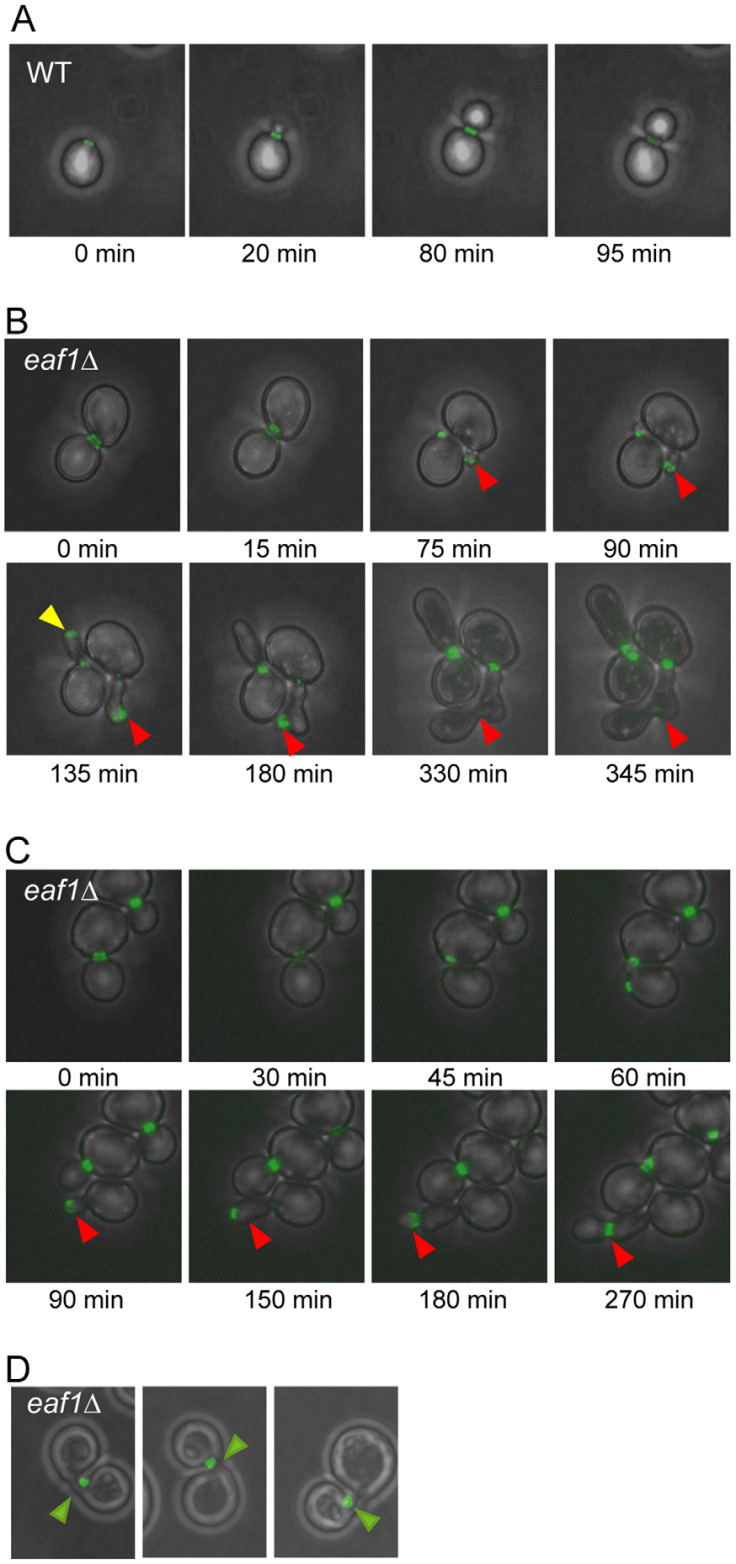
*eaf1Δ* cells have defects in septin dynamics. Time lapse imaging of cells expressing Cdc11-GFP in the presence (WT, YKB1312) or absence of *EAF1* (*eaf1Δ,* YKB1310). Cells were grown to mid-log phase in YPD supplemented with adenine at 25°C, and transferred onto synthetic complete agarose pads for imaging at room temperature. Images were collected at five or fifteen minute intervals for wild type and *eaf1Δ* cells, respectively. Representative images of cells over time are shown in panel (**A**) for wild type cells and panels (**B–C**) for *eaf1Δ* cells. Red arrows point to septins mislocalized into the bud. The yellow arrow points out initially mislocalized septins that re-localize to the bud neck, followed cell division. (**D**) Images of three different *eaf1Δ* cells exhibiting septins misorganized to one side of the neck. Quantification of live cell imaging is presented in Table 1.

As septin misorganization or mislocalization is one of the cellular defects that can activate the morphogenesis checkpoint [51], we next examined septin localization in NuA4 deletion mutants. During the cell cycle, septins adopt two predominant structures: a ring that assembles at the incipient bud site in early G1, and an hourglass-shaped collar that develops upon bud emergence. Just prior to cytokinesis, the collar splits into two rings that are inherited by the mother and daughter and disassembled following cytokinesis, thereby resetting the cycle [38], [47], [48]. The transition between the ring and collar relies on structural changes in the organization of hetero-octameric septin complexes at the bud neck, but the molecular basis is poorly understood [52]. To explore the role of NuA4 in septin regulation, using time-lapse fluorescence microscopy, we examined the localization of Cdc11-GFP in both wild type and *eaf1Δ* cells. In wild type cells, Cdc11-GFP localized as described above in all cells examined (Figure 3A and Table 1). However, we discovered a defect in septin localization in 24% of *eaf1Δ* cells in this analysis (Figure 3B, C, D and Table 1). The major defect, observed in 19% of *eaf1Δ* cells, occurred at the ring-to-collar transition, coinciding with bud emergence. Specifically, while formation of the septin ring appeared normal in these cells, rather than re-organizing into a collar structure upon bud emergence, Cdc11-GFP diffused into the bud. Next, Cdc11-GFP either assembled into a new ring at the bud tip and a new bud emerged, or it remained diffusely localized near or in the bud tip. In both cases the cells developed elongated buds, consistent with our observations above. Interestingly, in some of the *eaf1Δ* cells with septins mislocalized into the bud tip, the defect was corrected as Cdc11-GFP re-localized to the bud neck followed by collar splitting and cell division (Figure 3B). A second less prominent defect we observed in only 5% of *eaf1Δ* cells was the misorganization of Cdc11-GFP to one side of the bud neck upon bud emergence (Figure 3D); while bud morphology appeared largely normal, over time the Cdc11-GFP re-structured into a collar that spanned the bud neck, albeit lacking the crisp structure of wild type cells, and the cells eventually divided. We also found that 11% of *eaf1Δ* of cells failed to assemble a new septin ring one hour or more post-cell division (Table 1). While this suggests a possible defect in septin ring assembly, given the slow progression of *eaf1Δ* mutants through the cell cycle in this experiment (time to complete cell division ranged from ∼3 to 6 hours), it cannot be ruled out that that these cells would have eventually developed a new septin ring. As overexpression of *CDC11* and *SHS1* impaired the fitness of almost every non-essential NuA4 deletion mutant (Figure 1C), we also examined Cdc11-GFP localization in the remaining six non-essential NuA4 deletion mutants and found that only *yng2Δ* mutants exhibited defects similar to *eaf1Δ* cells (Figure S1). As Yng2 is the only other non-essential subunit required for Esa1 catalytic activity *in vivo* [9], this work indicates that NuA4 acetyltransferase activity is required for faithful septin dynamics, in particular during the re-organization of septin filaments into the collar upon bud emergence.

**Table 1:** Quantification of defects in *eaf1Δ* cells.

Morphology		WT	*eaf1Δ*
Bud	Septin	(n = 15)	(n = 43)
Normal	Normal	100%	65%
Elongated	Mis-localized in bud	0%	19%
No bud	Ring did not re-assemble (>1 hour)	0%	11%
Normal	Localized to one side of neck	0%	5%

Using time lapse imaging of cells expressing Cdc11-GFP in the presence (WT (YKB1312)) or absence of *EAF1* (*eaf1Δ* (YKB1310)), mother cells (immediately after cytokinesis) were followed through a cell cycle and bud and septin morphology was scored. Images were collected at five or fifteen minute intervals for wild type and *eaf1Δ* cells, respectively, at room temperature.

### NuA4 regulation of septin dynamics occurs independently of the histone H4 N-terminal tail

**Figure 4:**
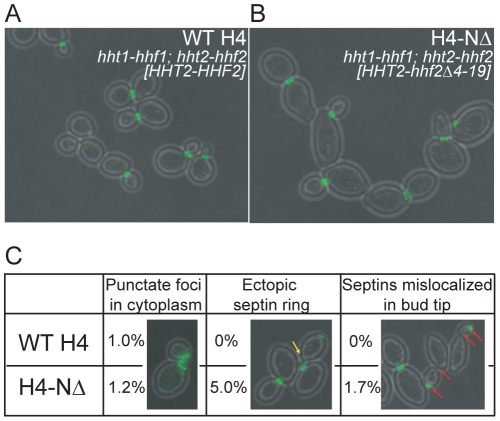
Septin localization defects of *eaf1Δ* cells are distinct from histone H4 tail mutants. Cells expressing Cdc11-GFP, in which the sole source of histone H4 was expressed from a plasmid encoding a wild type (WT H4, YKB1260) or a mutant gene (H4-NΔ, YKB1261), were grown to mid-log phase at 25°C in YPD medium supplemented with adenine. Cells were fixed with paraformaldehyde prior to imaging by fluorescence microscopy. H4-NΔ encodes a protein in which amino acids 4 to 19 are deleted. Representative images of WT H4 and H4-ΔN cells are shown in (**A**) and (**B**), respectively. The yellow arrow points to an ectopic septin ring and the red arrows point to cells with septin in both the bud tip and neck. (**C**) Quantification of the defects observed for Cdc11-GFP localization in the cells represented in (A) and (B). The experiment was performed in triplicate scoring more than 100 cells in each replicate and the average is presented.

It was previously reported that both Esa1 and the N-terminal tail of histone H4 become essential when the morphogenesis checkpoint is constitutively activated [53], [54]. This suggests the basis of these genetic interactions might lie in altered chromatin structure or transcriptional misregulation derived from defects in histone H4 modifications. To test the hypothesis that histone H4 acetylation by NuA4 controls septin dynamics *in vivo*, we examined Cdc11-GFP localization and bud morphology in cells expressing a plasmid-borne copy of wild type or N-terminal deletion mutant (amino acids 4 to 19) (H4-ΔN) as the sole source of histone H4 [55]. We discovered that H4-ΔN cells were strikingly more oblong in both mothers and buds than their wild type counterparts (Figure 4A and B), however, in the vast majority of mutants cells (98.3%), septins localized to the bud neck as in wild type (100%). We did observe a few cells with ectopically localized Cdc11-GFP in addition to the correctly formed ring or collar, but in only 1.7% of H4-ΔN cells were septins mislocalized to the bud tip (Figure 4C). As the phenotypes we observed in H4-ΔN mutants were largely distinct from *eaf1Δ* mutants, this suggests NuA4 is not regulating septin dynamics and bud morphology through the acetylation of the N-terminal tail of histone H4 or altered chromatin structure. As NuA4 acetylates the histone H2A variant Htz1, we also examined Cdc11-GFP localization in cells lacking *HTZ1* and discovered these cells were indistinguishable from their wild type counterpart (data not shown). Together, these data indicate that the role of NuA4 in septin dynamics occurs independently from its primary histone acetylation targets, potentially through the acetylation of non-histone proteins.

### Septin proteins are acetylated *in vivo* and *in vitro* by NuA4

**Figure 5:**
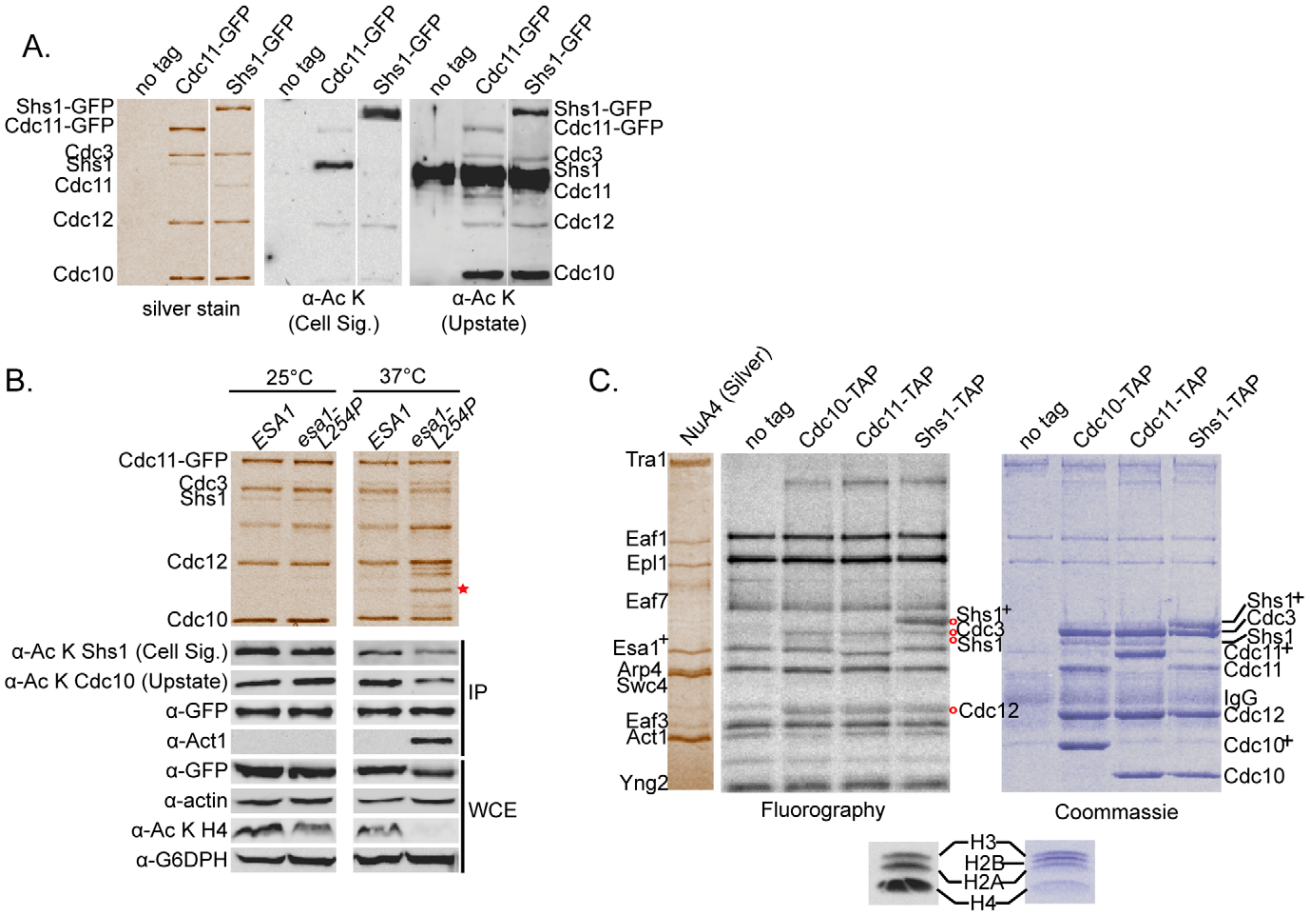
Septin proteins are acetylated *in vivo* and *in vitro*. (**A**) Cdc10 and Shs1 are acetylated *in vivo*. Septin protein complexes were immunopurified through the GFP tag and separated by SDS-PAGE (7.5%) in cells expressing Cdc11-GFP (YKB1312), Shs1-GFP (YKB1878), or an untagged strain (YKB780; no tag). Left: silver stain to indicate the relative migration of septins. Right: Western blots using anti-acetyl lysine (α-Ac K) antibodies from Cell Signaling (Cell Sig.) or Upstate. Intervening lanes are cropped. (**B**) Acetylation of Shs1 and Cdc10 is dependent on Esa1 KAT activity *in vivo*. Septin proteins were immunopurified from cells grown at 25°C or 37°C for four hours through Cdc11-GFP in a wild type *ESA1* (YKB1312) or mutant *esa1-L254P* background (YKB1804). Immunopurified (IP) products and whole cell extract (WCE) samples were separated by SDS-PAGE, silver stained or subjected to Western blot analysis. Shs1 and Cdc10 acetylation was assessed using antibodies in (A). Expression and immunopurification of Cdc11-GFP and Act1 was assessed with α-GFP and α-actin antibodies. Esa1 catalytic activity was assessed through histone H4 acetylation levels (α-Ac K H4). Glyceraldehyde-6-phosphate dehydrogenase (α-G6PDH) is a loading control. Red star: actin. This figure is representative of five experimental replicates. Intervening lanes are cropped. (**C**) NuA4 acetylates septins *in vitro*. *In vitro* KAT assays were carried out on septins immunopurified through Cdc10-TAP (YKB1443), Cdc11-TAP (YKB1455), or Shs1-TAP (YKB1466), relative to an untagged control (YKB779; no tag) using NuA4 purified from yeast and [^3^H]-acetyl coenzyme A. Reactions were separated by SDS-PAGE (7.5%), visualized by Coomassie staining, treated for fluorography, and visualized on film. The migration of NuA4 subunits is shown on the left. Acetylation of chicken core histones monitors NuA4's activity *in vitro*. Plus sign: shift in mobility on the gel due to the presence of the calmodulin binding peptide. Red circles: septin proteins that are acetylated *in vitro*. This figure is representative of two experimental replicates.

As other studies have shown that SDL screens can identify targets of kinases [35], [37], the SDL interactions between NuA4 mutants and *CDC11* and *SHS1* encouraged us to directly test whether one or more septin proteins are acetylated *in vivo*. To this end, septin protein complexes were purified through endogenously tagged Cdc11-GFP and Shs1-GFP. Septin hetero-octamers contain two each of Cdc3, Cdc10, and Cdc12, and further one or two of Cdc11 and/or Shs1 [56]. Western blot analysis of the isolated septins using two different anti-acetyl lysine antibodies suggested that Cdc10 and Shs1 are acetylated *in vivo* (Figure 5A). While both antibodies recognized Shs1, only one recognized Cdc10, indicating the possibility of variability in epitope recognition. Notably, considering the ratio of the acetylation signal to the quantity of protein purified (assessed by silver stain) the Shs1-GFP protein was much less acetylated in comparison to the untagged Shs1 protein that co-purified through Cdc11-GFP. (Figure 5A, α-Ac K Cell Sig.). To assess the *in vivo* dependence of septin acetylation on NuA4 activity, we evaluated the acetylation status of Shs1 and Cdc10 through Western blot in strains harboring either a wild type or thermosensitive *ESA1* allele, *esa1-L254P* mutant [11]. The protein encoded by *esa1-L254P* is catalytically inactive at the restrictive temperature of 37°C [11]. Following immunopurification through Cdc11-GFP, we discovered that while acetylation of Cdc10 and Shs1 was unchanged at 25°C, a reduction in acetylation on both proteins was observed specifically in the *esa1-L254P* strain background in cells grown at 37°C (Figure 5B). This result indicates that acetylation of both Cdc10 and Shs1 is partially dependent on Esa1 *in vivo*. Supporting the possibility that additional KATs may regulate septin dynamics through the acetylation of septin proteins, we have discovered that two additional yeast KAT mutants, *gcn5Δ* [57] and *eco1-203* [58], also have defects in septin localization, although not as severe as those observed in *eaf1Δ* cells (Figure S2). Interestingly, through mass spectrometry we discovered that components of the actin cytoskeleton, including actin itself (Act1) and two members of actin cortical patches, Abp1 and Crn1 [59], co-purify with Cdc11-GFP in the *esa1-L254P* strain background at 37°C. Co-purification of Act1 with septins was also detected in the acetyltransferase-deficient *eaf1Δ* and *yng2Δ* strain backgrounds (data not shown). To specifically examine the ability of NuA4 to acetylate septin proteins, we performed *in vitro* NuA4 KAT assays on septins immunopurified from yeast (Cdc10- Cdc11- and Shs1-TAP) using radiolabeled acetyl coenzyme A. This analysis confirmed that in addition to acetylating itself, NuA4 was capable of acetylating Cdc3, Cdc12 and Shs1 *in vitro*, however acetylation on Cdc10 was not detected (Figure 5C). As septins were immunopurified from wild type yeast cells, it is possible that Cdc10 was fully acetylated prior to the assay, thereby precluding additional *in vitro* acetylation by NuA4. Finally, to identify the modified lysine residues, septins were purified through endogenously TAP-tagged Cdc11-TAP or Shs1-TAP, and mass spectrometry was performed. To enrich acetylation levels within the immunopurified septins, half of each immunopurified sample was subjected to an *in vitro* KAT reaction using highly purified NuA4 [13] and acetyl coenzyme A. Eighteen lysine acetylation sites on four septin proteins (Table 2 and Table S5), as well as N-terminal acetylation sites on all five septins (Table S5) were identified. While a few acetyl lysine sites were unique to the NuA4 *in vitro* KAT assay samples (Cdc3 K316; Cdc12 K193 and K338; Shs1 K82, K443, K488), the rest were also identified in the untreated samples, confirming that septins are acetylated *in vivo*. In summary, these data demonstrate that at least four of the five septin proteins are acetylated *in vivo*, implicate NuA4 in the acetylation of Shs1 both *in vitro* and *in vivo*, and indicate Cdc10 acetylation relies on Esa1 activity *in vivo*.

**Table 2:** Acetylated lysine residues on septin proteins.

Septin	Protein Coverage	Acetylation site
Cdc3	77%	K4, K137, K316*
Cdc10	60%	K73, K78, K128, K166
Cdc11	73%	----
Cdc12	81%	K193*, K251, K338*
Shs1	62%	K57, K82*, K204, K352, K443*, K478, K488*, K536

* =  Acetylation site was only identified in sample subjected to NuA4 *in vitro* KAT assay.

### Unacetylable shs1 mutants have bud morphology and septin localization defects similar to NuA4 mutants

**Figure 6:**
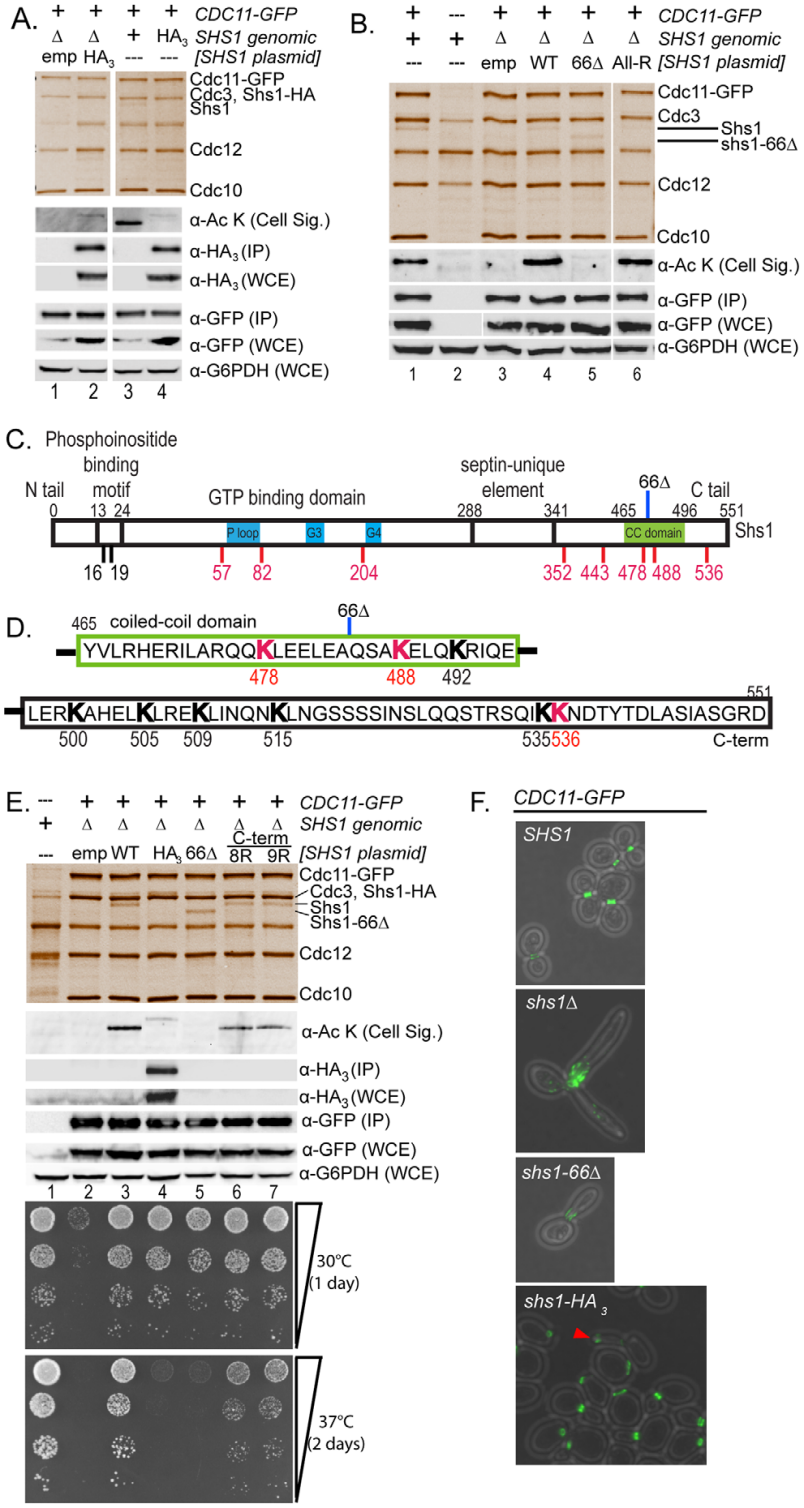
Unacetylable Shs1 mutants display defects in bud morphology and septin localization. (**A**) Shs1-HA_3_ exhibits decreased acetylation *in vivo*. Immunopurified septins were analyzed as in Figure 5. Lanes 1 and 2: Cells expressing *CDC11-GFP* and lacking genomic *SHS1* (Δ) transformed with an empty vector (emp, YKB2254), or plasmid encoding *SHS1-HA_3_* (HA_3_, YKB2255). Lanes 3 and 4: Cells expressing genomic *CDC11-GFP* and *SHS1* as an untagged protein (+, YKB1312) or *SHS1–HA_3_* fusion protein (HA_3_, YKB2518). Antibodies are described in Figure 5. (**B**) C-terminal truncation abolishes Shs1 acetylation. Lane 1: Cells expressing genomic *CDC11-GFP* and *SHS1* (YKB 1312). Lane 2: Untagged control (YKB780). Lanes 3–6: Cells expressing *CDC11-GFP* and lacking *SHS1* (Δ) transformed with an empty vector (emp, lane 3, YKB2519), plasmid encoding wild type *SHS1* (WT, lane 4, YKB2520), C-terminal *shs1* truncation (66Δ, lane 5, YKB2521), or *shs1* lysine-to-arginine mutant (All-R: K16,K19,K57,K82,K204,K443,K478,K488,K536, lane 6, YKB2523). Intervening lanes are cropped. (**C and D**) Schematic outlining Shs1 domains and acetylation sites. Domains are noted on top in black. Acetylated lysine residues identified by mass spectrometry are in red along the bottom. Additional lysines used in mutational analyses are in black. 66Δ: site of C-terminal Shs1. (**E**) C-terminal Shs1 lysine mutations reduce *in vivo* acetylation and cell fitness. Lane 1: untagged control (YKB780). Lanes 2-7: Cells expressing *CDC11-GFP* and lacking *SHS1* (Δ) transformed with an empty vector (emp, lane 2, YKB2519), plasmid encoding wild type *SHS1* (WT, lane 3, YKB2520), C-terminal *shs1* truncation (66Δ, lane 4, YKB2521), C-terminal *shs1* lysine-to-arginine point mutants (8R: K488,K492,K500,K505,K509,K515,K535,K536, lane 6, YKB2747; 9R (8R + K478 lane 7, YKB2748). Representative of three experimental replicates. Bottom panels: strains spotted in ten-fold serial dilutions on YPD plates. (**F**) Bud morphology and septin localization in *shs1* mutants. Cells were grown at 30°C and fixed. Genomic *SHS1* was expressed as wild type (*SHS1;* YKB2712), C-terminal truncation (*shs1-66*Δ; YKB2713), fusion protein (*SHS1-HA_3_*; YKB2518), or deleted from the genome (*shs1*Δ; YKB2173). Red arrow: mislocalized septins within an elongated bud.

We next sought to establish a direct cause-and-effect relationship between septin acetylation and the defects in septin organization and localization observed in NuA4 mutants. To this end we tested whether loss of septin acetylation, achieved by mutating acetylated lysine residues on septin proteins to arginine, could re-capitulate the phenotypes observed in the NuA4 mutants. We focused our efforts on Shs1 for multiple reasons. First, previous studies have established the use of SDL screens for the identification of enzyme-substrate relationships [35], [37], [60]; as overexpression of *SHS1* caused SDL phenotypes in NuA4 non-essential deletion mutants, this predicts that Shs1 is a target of NuA4. In agreement with this, we found that Shs1 is the most abundantly acetylated septin protein as detected by mass spectrometry (Table 2), and a target of NuA4 both *in vitro* and *in vivo* (Figure 5). We initially cloned a C-terminally epitope-tagged version of *SHS1* (*SHS1-HA_3_*; encoding three copies of the hemagglutinin epitope) into a low copy plasmid under the control of the endogenous *SHS1* promoter. However, when septin protein complexes were purified through Cdc11-GFP in exponentially growing cells, we discovered that the Shs1-HA_3_ fusion protein, whether expressed from a plasmid or the endogenous genomic locus (Figure 6A, lanes 2 and 4, respectively), was acetylated to only a small degree *in vivo* relative to the untagged, endogenously encoded Shs1 protein (Figure 6A, lane 3). As Shs1-HA_3_ co-migrated with Cdc3, its incorporation into the septin hetero-octamer was assessed by Western blot. By eliminating the HA_3_ epitope tag from the *SHS1*-encoding plasmid, acetylation of Shs1, immunopurified through Cdc11-GFP, could be restored to the level observed on the genomically encoded Shs1 (Figure 6B; lanes 1 and 4). This result suggested that the presence of the relatively small HA_3_ tag (∼3 kDa, 30 amino acids) effectively blocked acetylation of Shs1 *in vivo* as detected in this Western blot analysis. Given the C-terminal location of the HA_3_ tag, this also indicated that despite the identification of acetyl lysine residues over the length of Shs1 (Table 2, Figure 6C), the predominant site(s) of acetylation might reside close to the carboxy terminus (Figure 6D).

To test the role of the C-terminal tail in Shs1 acetylation, we subsequently generated a C-terminal truncation in the *SHS1* plasmid by deleting the nucleotide sequence encoding the final 66 amino acids of Shs1 (*shs1-66Δ*) (Figure 6C and D). In total, the truncation eliminated eight lysine residues, two of which were identified as acetyl lysine residues by mass spectrometry (K488 and K536; Table 2). We discovered that despite incorporation of truncated Shs1 (shs1-66Δ) into the Cdc11-GFP immunopurified septin protein complex to the same degree as wild type Shs1 (Figure 6B silver stain, compare lanes 4 and 5), the acetylation signal of shs1-66Δ was completely abolished (Figure 6B, α-Ac K Western blot, lanes 4 and 5). This suggested that Shs1 acetylation indeed relies on the C-terminal tail. To assess the contribution of acetyl lysine residues identified by mass spectrometry to the overall acetylation status of Shs1, we generated a multi-site *shs1* mutant (‘All-R’), by mutating the majority of these sites (K57, K82, K204, K352, K443, K478, K488, K536) plus two additional N-terminal lysines (K16, K19) to arginine. The resulting signal on the ‘All-R’ mutant was unchanged relative to wild type Shs1, indicating that these lysines do not contribute substantially to the acetylation of Shs1 immunopurified through Cdc11-GFP (Figure 6B, lane 6). To specifically test the contribution of C-terminal lysine residues in Shs1 acetylation, we generated Shs1 plasmids with lysine-to-arginine point mutations in the eight (8R: K488, K492, K500, K505, K509, K515, K535, K536) or nine (9R: 8R + K478) C-terminal most lysine residues. While the All-R mutant indicated that K478, K488, or K536 are not the exclusive sites of acetylation in the C-terminus, we included these mutations to account for the possibility of functional redundancy or partial contribution. Septin protein complexes were subsequently immunopurified through Cdc11-GFP and the acetylation status of co-purified Shs1 assessed by Western blot. In each of the 8R and 9R mutants we observed a clear and reproducible reduction in acetylation on Shs1 (Figure 6E, lanes 6 and 7) as compared to the wild type Shs1 (Figure 6E, lane 3). However, the drop in signal was neither complete, as in the shs1-66Δ mutant (Figure 6E, lane 5), nor as marked as the reduction observed on the Shs1-HA_3_ fusion protein (Figure 6E, lane 4). If Shs1 acetylation, as detected by this antibody, occurred exclusively within the C-terminal tail, then the signal on the 8R and 9R mutants should have been completely ablated. Together, these results suggest that the primary site(s) of acetylation do not reside in the C-terminus, but that maximal acetylation of Shs1 depends on the presence of an unperturbed C-terminal tail. In agreement, cells expressing the 8R or 9R C-terminal Shs1 mutants were only moderately temperature sensitive as compared to near lethality of those expressing Shs1-HA_3_ or shs1-66Δ (Figure 6E).

Given the loss of acetylation in the *shs1-66Δ* and *shs1-HA_3_* mutants, we wondered whether these cells would display septin defects similar to those observed in NuA4 acetyltransferase mutants. Thus, using fluorescence microscopy, we examined bud morphology and septin localization in cells expressing Cdc11-GFP in which the genomic copy of *SHS1* was intact (*SHS1*), fully deleted (*shs1Δ*), C-terminally truncated (*shs1-66D*), or fused to an HA_3_ tag (*shs1-HA_3_*). As expected, cells expressing wild type *SHS1* exhibited normal bud morphology and septin localization in all cells examined (Figure 6F, *SHS1*). On the other hand, 100% of cells completely lacking *SHS1* displayed highly elongated, multi-budded cell morphology, with severely fragmented Cdc11-GFP signal throughout the cell but concentrated near the bud neck(s) (Figure 6F *shs1Δ*). While the majority of cells in the *shs1-66Δ* population exhibited normal bud and septin morphology, in 40% of these mutant cells we observed highly elongated buds in which septins were misorganized at the bud neck in a specific pattern previously described as resembling ‘parallel bars’ [49] (Figure 6F *shs1-66Δ*). Though acetylation was eliminated from the truncated shs1-66Δ protein, this phenotype did not correspond to the defects observed in *eaf1*Δ mutant cells (Figures 3B, C, and D). Shs1 is heavily post-translationally modified by both phosphorylation [61] and sumoylation [62] in its C-terminus and a role for the C-terminal domain of Shs1 has been suggested for septin structural transitions [56]. We hypothesize that the phenotype observed in the *shs1-66Δ* mutant might be attributable to a combination of these factors. To specifically examine the consequence of loss of Shs1 acetylation in an intact protein, we finally examined the bud morphology and septin localization in cells expressing *SHS1-HA_3_*, on which acetylation is virtually eliminated but in which the C-terminal tail is intact (Figure 6A and 6E). We discovered that cells expressing the relatively unacetylable Shs1-HA_3_ protein had a phenotype similar to *eaf1Δ* mutants, in that 5% of cells examined displayed elongated buds with septins mis-localized to the bud tip (Figure 6F, *SHS1-HA_3_*, red arrows). Importantly, as observed in *eaf1Δ* mutants, all small, unbudded cells contained a correctly formed septin ring, suggesting that loss of Shs1 acetylation does not impact this stage of septin organization. Although 5% is a relatively small proportion of cells, this phenotype is never observed in wild type cells and as such represents an important observation. If the sole consequence of fusing an HA_3_ tag to the C-terminus of Shs1 is to reduce the level of acetylation on the protein *in vivo* (see discussion), this result lends support to the hypothesis that the acetylation of Shs1 directly impacts septin dynamics.

## Discussion

In this work, we performed genome-wide SDL screens to assess the function of the NuA4 lysine acetyltransferase complex and uncovered a novel role for NuA4 in septin dynamics. Global SDL profiling represents a relatively new addition to the yeast functional genomics toolkit. To date, genome-wide SDL screens have been applied to assess the function of three kinases [35], [37], and to explore the function of a particular proteolytic degradation pathway [60]. In agreement with the long-standing observation that SDL interactions occur between members of the same pathway [63], global SDL screens have successfully identified substrates of kinases and the work we present here indicates this will hold true for predicting the substrates of KATs. In principle, NuA4 SDL and SL interactions may now be utilized in a complementary fashion, with SL interactions linking NuA4 to cellular processes and SDL interactions predicting the specific pathways in which the complex functions. Interestingly, a comparison of the number of NuA4 SDL interactions uncovered in this study (89) to previously published NuA4 genome-wide SL interactions (>500) (Table S4), suggests that while NuA4 function buffers a diverse array of cellular processes, it may directly function in only a handful of pathways.

Here we show for the first time a link between KAT function and the regulation of septin dynamics (Figure 3; Figure S2). Specifically, our time-lapse imaging implicates NuA4 function in the septin structural transition that occurs upon bud emergence, as some *eaf1Δ* cells, though capable of assembling a septin ring at the incipient bud site, failed to re-assemble septins into a collar. The most prominent defect observed was the mislocalization of septins within elongated bud tips, while a minority of cells exhibited septins misorganized to one side of the bud neck. That we also observed septin mislocalization within elongated buds in *eco1* and *gcn5* mutants cells (Figure S2) suggests common or redundant regulatory roles for KATs in some aspect of septin dynamics. These observations support the addition of NuA4 and other KATs onto the list of proteins already implicated in the ring-to-collar structural transition, including Cdc42, Cla4, Elm1, Gin4, Bni5, Bud3, Bud4, Bud5, Bni1, Cln1, and Cln2 [49], [64], [65]. In general, the ring-to-collar transition is poorly understood mechanistically as it is likely regulated by multiple parallel pathways. The fact that NuA4 mutants (*esa1-L254P*, *eaf1Δ*) display synthetic sickness with *cla4Δ* mutants [13], [66] suggests that NuA4 and Cla4 may work in parallel to promote septin organization. Importantly, in all cases, similar to *eaf1Δ* cells, mutations in these genes result in phenotypes with incomplete penetrance [49], [67], [68].

How do NuA4 and other KATs regulate septin dynamics? In this study we have explored the hypothesis that one pathway involves the direct acetylation of septin proteins. We have demonstrated that four septin proteins are acetylated *in vivo* (Table 2), that NuA4 is capable of acetylating three septin proteins *in vitro* (Figure 5C) and that the acetylation of at least Cdc10 and Shs1 depend in part on Esa1 function *in vivo* (Figure 5B). While we did not identify acetyl lysine residues on Cdc11, given the interchangeable role described for Cdc11 and Shs1 as the outermost subunit within hetero-octameric septin protein complexes [56] it is possible that acetylation sites may still be mapped onto this subunit. Our discovery that septin proteins are acetylated adds an additional layer of complexity to an already crowded roster of post-translational modifications (PTMs) on yeast septin proteins. A plethora of phosphorylation and sumoylation sites have been identified on multiple septin proteins through the action of a handful of kinases and one SUMO-conjugating enzyme [52]. Notably, the biological consequences of these modifications, which have generally proven difficult to dissect, have by-and-large been linked to septin structural transitions [61], [64], [69], [70], [71].

To establish a direct cause-and-effect relationship between septin acetylation and KAT function, we carried out a mutational analysis of Shs1. Importantly, we have demonstrated that the elongated bud and septin mislocalization phenotypes observed in acetyltransferase-deficient NuA4 mutant *eaf1Δ* cells (Figure 3B–D) can be partially re-capitulated in cells expressing Shs1-HA_3_, which displays drastically reduced acetylation levels (Figure 6F). A number of factors could explain the relatively small effect on septin localization and bud morphology observed in the Shs1-HA_3_ fusion mutant. First, given the epitope variability of anti-acetyl lysine antibodies, (Figure 5A), it is possible the acetylation signal detected on Shs1 by the Cell Signaling antibody does not recognize the full complement of acetylation on Shs1. Hence, though acetylation as detected by Western analysis appears diminished, additional acetyl lysine residues may still be present on the Shs1-HA_3_ protein. An alternative, but not mutually exclusive, explanation is that acetylation on other septin subunits contributes to this phenotype. We also acknowledge that other interpretations of the phenotype associated with the loss of acetylation on Shs1-HA_3_ exist. For instance, fusion of the HA_3_ tag may also interfere with PTM's such as phosphorylation and sumoylation on Shs1, thereby mediating the phenotype, which we hypothesize is the case for the *shs1-66Δ* mutant (Figure 6F and text). Alternatively, while Western blot analysis indicates that Shs1-HA_3_ is incorporated into the septin hetero-octamer (Figure 6A lanes 1 and 3, Figure 6E lane 4), as the fusion protein co-migrates with Cdc3, whether it incorporates to the same level as untagged Shs1 cannot be determined from the silver stain. Thus, the reduced acetylation signal on Shs1-HA_3_ may be a function of reduced co-purification with Cdc11-GFP, and the loss of incorporation into the septin complex may underlie the phenotype. In the absence of generating Shs1-directed antibodies, it is difficult to distinguish these possibilities. However, as the absence of the C-terminal tail on the shs1-66Δ does not affect incorporation of the mutant protein into the hetero-oligomer (Figure 6B and E, lane 5 in both), and as the interaction of Shs1 with the septin hetero-octamer has previously been shown to occur through its GTP-binding domain [46], we do not believe this to be a strong possibility.

What biological role does Shs1 acetylation play? As the acetylation-incompetent Shs1 mutant proteins co-immunopurify via Cdc11-GFP (Figure 6B and 6E), this suggests that Shs1 acetylation does not specifically regulate hetero-octamer assembly. Another possibility is that Shs1 acetylation impacts some aspect of higher-order septin assembly, achieved through end-on-end polymerization of septin hetero-octamers. That septin rings form normally in unbudded *eaf1*Δ cells or cells expressing Shs1-HA_3_ suggests that the specific higher-ordered assembly of hetero-octamers at this stage of the cell cycle is not impacted by loss of Shs1 acetylation. On the other hand, our data suggest that both *eaf1*Δ cells (Figure 3B and C) and Shs1-HA_3_ expressing cells (Figure 6) display defects in establishing the septin collar, indicating that either assembly or stabilization of the specific septin polymers required for the ring-to-collar transition are impaired [46]. Though the exact role of Shs1 in septin dynamics is likely pleiotropic, it has been suggested that Shs1 serves as a cap to hetero-oligomer polymerization rather than propagating assembly of filaments [46]. If acetylation is required for a capping function of Shs1, in the absence of acetylation, uninhibited polymerization may lead to septin mislocalization [46]. Similarly, phosphorylation of Shs1 has previously been linked to the dynamic behavior of septins [72], however the exact molecular impact of these PTMs has yet to be established.

Our work provides the first evidence that KAT function regulates septin dynamics and is the first characterization of acetylation on septin proteins. Dissecting the functional consequences of acetylation presents many challenges, including identifying the biologically relevant sites of acetylation, assessing temporal changes, and assigning KATs to each site. Importantly, septin acetylation has also been identified in high throughput proteomic studies in human cells [4], [5]. Intriguingly, at least one component of the human homolog of NuA4, the Tip60 complex, is known to localize to the cytokinetic furrow during mitosis [73], suggesting that KAT regulation of septin dynamics and septin acetylation is highly conserved. However, our data also indicate that NuA4 likely impacts additional pathways related to septin proteins. For instance, in the NuA4 SDL map we identified a number of genes involved in some aspect of cytokinesis, a process intimately linked to septin dynamics. This includes two chitin synthase enzymes, Chs2 and Chs3, that function at the primary septum and throughout the cell wall respectively [74], [75]; the multifunctional protein phosphatase 1, Glc7, and its regulator Bni4, that together localize Chs2 to the bud neck [76]; and a number of proteins involved in both formation and regulation of the actin cortical patch and cytoskeleton, Arc40, Rvs167, Akl1, Sla1, and the actin structural molecule itself, Act1 [59]. An alternative and intriguing role for NuA4-dependent acetylation is that it may lead to changes in protein-protein interactions. Indeed, we have discovered that KAT-deficient NuA4 mutants alter physical interactions between septin proteins and components of the actin cytoskeleton (Figure 5B). More broadly, acetylome studies [2], [4], [5], [77] clearly indicate that lysine acetylation is an abundant PTM, hence it is possible that numerous pathways in addition to septin proteins may be regulated by NuA4-dependent acetylation to ultimately regulate septin dynamics and the successful completion of cytokinesis.

## Supporting Information

Figure S1 Cdc11-GFP localization in six non-essential NuA4 mutants. Cells expressing Cdc11-GFP, in which one non-essential NuA4 gene was deleted (*eaf3Δ* (YKB1376); *eaf5Δ* (YKB1378); *eaf6Δ* (YKB1379); *eaf7Δ* (YKB1381); *yaf9Δ* (YKB1383); *yng2Δ* (YKB1385)) were grown to mid-log phase at 25°C in YPD medium supplemented with adenine. Cells were fixed with paraformaldehyde prior to imaging by fluorescence microscopy. Representative images in each stage of the cell cycle are presented. More than 200 cells were examined for each mutant.
(TIF)

Figure S2 Cdc11-GFP localization in KAT mutants. (**A**) Bud morphology and septin localization in KAT mutant strains grown to mid-log phase at 25°C in YPD medium supplemented with adenine. Strains include: *rtt109Δ* (YKB1672); *sas2Δ* (YKB1668); *hat1Δ* (YKB1682); *hpa2Δ* (YKB1679); *elp3Δ* (YKB1676); *sas3Δ* (YKB1800); *spt10Δ* (YKB1796); *gcn5Δ* (YKB1664); *eco1-203* (YKB2145); *eaf1Δ* (YKB1310)). Cells were fixed with paraformaldehyde prior to imaging by fluorescence microscopy. At least hundred cells were counted for each strain. (**B**) *GCN5*, and *ECO1* mutants have defects in cell morphology and septin localization. Representative images from (A) are shown.
(TIF)

Table S1 Strains used in this study.
(DOCX)

Table S2 Plasmids generated for this study.
(DOCX)

Table S3 NuA4 SDL Interactions. Detailed scoring of all SDL and SDS genetic interactions identified for the seven non-essential NuA4 mutants (*eaf1Δ* (YKB44); *eaf3Δ* (YKB1162); *eaf5Δ* (YKB658); *eaf6Δ* (YKB504); *eaf7Δ* (YKB530); *yaf9Δ* (YKB464); *yng2Δ* (YKB494)). Overexpression of all interacting genes was also tested in a wild type background strain (WT (YKB779)). The scoring legend is at the bottom of the table.
(XLS)

Table S4 NuA4 SL Interactions. A list of published SL genetic interactions for the five non-essential NuA4 subunits subjected to genome-wide SDL screening (*EAF1, EAF3, EAF5, EAF6, EAF7*). Genes included in the list display an SL interaction with one or more of these five non-essential NuA4 genes. Interactions were downloaded from Biogrid (http://thebiogride.org/) May 2011.
(XLS)

Table S5 Acetylated lysine residues on yeast septin proteins. LC-MS/MS was used to identify acetylation sites on peptides derived from septin proteins. N-terminal acetylation is indicated by ‘N-term’. Treatment refers to whether the immunopurified septin proteins were subjected to a NuA4 *in vitro* KAT reaction with acetyl coenzyme A prior to LC-MS/MS. Acetylation sites identified in treated samples are indicated by ‘+ acetyl coA’. Samples were processed on one of two different mass spectrometers as indicated in the Instrument column. All acetylated peptides identified in multiple experiments are listed with their respective peptide scores.
(XLS)
