# Supplementary figures and images for: Regulation of Septin Dynamics by the Saccharomyces cerevisiae Lysine Acetyltransferase NuA4
Source: PLoS One. 2011 Oct 3;6(10):e25336. doi: 10.1371/journal.pone.0025336 (PMC3184947; doi:10.1371/journal.pone.0025336)

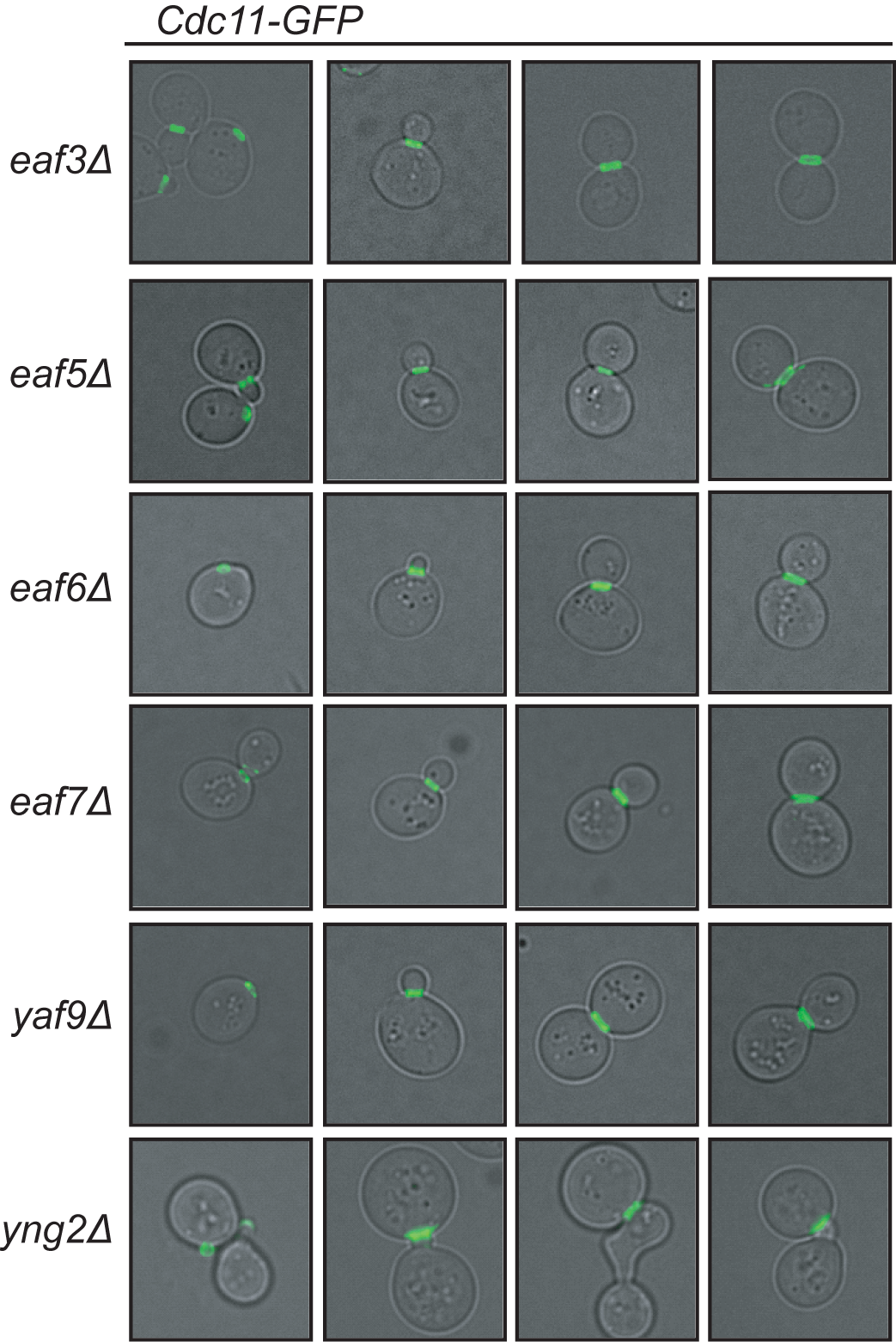

Supplement: Figure S1 — Cdc11-GFP localization in six non-essential NuA4 mutants. Cells expressing Cdc11-GFP, in which one non-essential NuA4 gene was deleted (eaf3Δ (YKB1376); eaf5Δ (YKB1378); eaf6Δ (YKB1379); eaf7Δ (YKB1381); yaf9Δ (YKB1383); yng2Δ (YKB1385)) were grown to mid-log phase at 25°C in YPD medium supplemented with adenine. Cells were fixed with paraformaldehyde prior to imaging by fluorescence microscopy. Representative images in each stage of the cell cycle are presented. More than 200 cells were examined for each mutant. (TIF) [file pone.0025336.s001.tif]

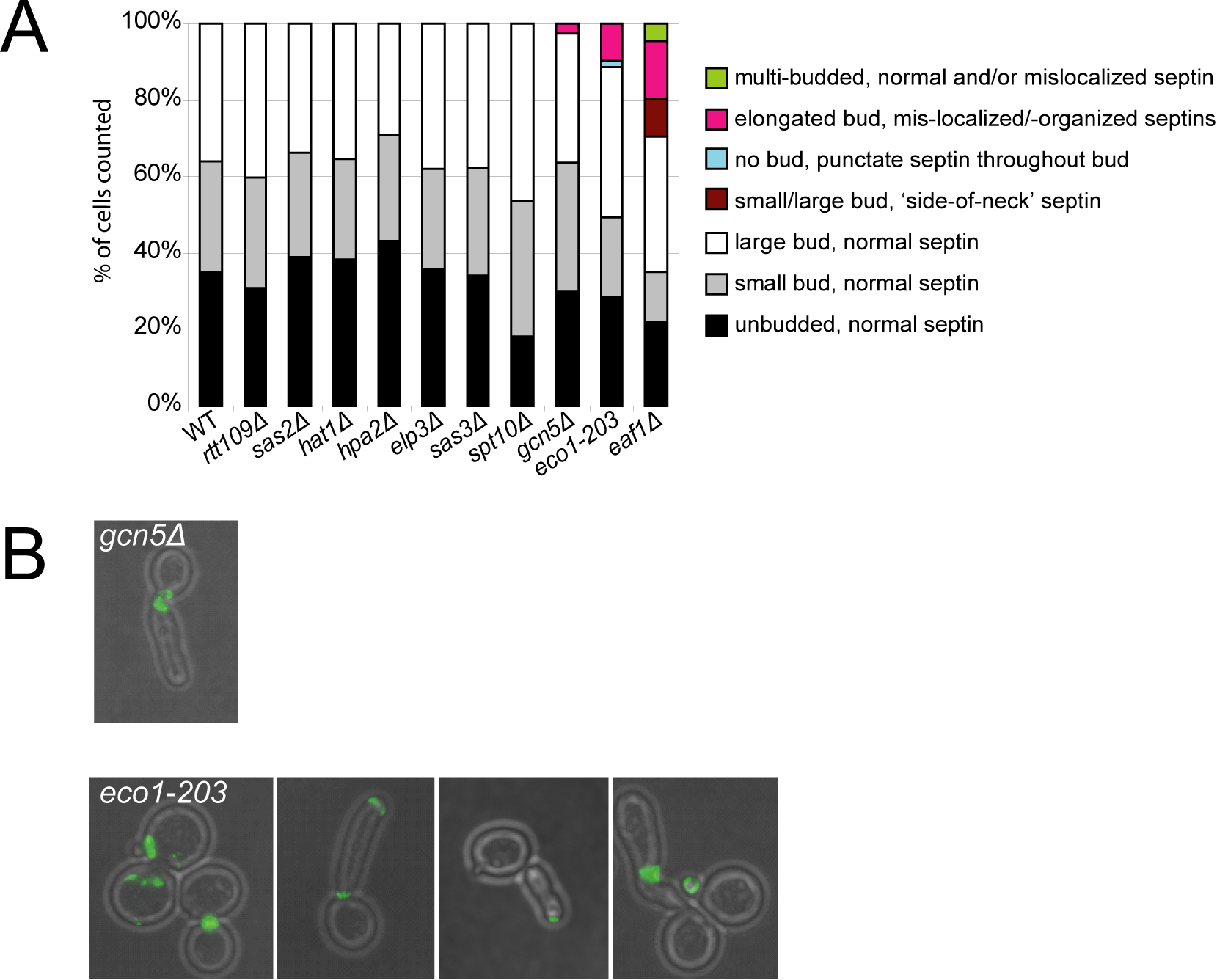

Supplement: Figure S2 — Cdc11-GFP localization in KAT mutants. (A) Bud morphology and septin localization in KAT mutant strains grown to mid-log phase at 25°C in YPD medium supplemented with adenine. Strains include: rtt109Δ (YKB1672); sas2Δ (YKB1668); hat1Δ (YKB1682); hpa2Δ (YKB1679); elp3Δ (YKB1676); sas3Δ (YKB1800); spt10Δ (YKB1796); gcn5Δ (YKB1664); eco1-203 (YKB2145); eaf1Δ (YKB1310)). Cells were fixed with paraformaldehyde prior to imaging by fluorescence microscopy. At least hundred cells were counted for each strain. (B) GCN5, and ECO1 mutants have defects in cell morphology and septin localization. Representative images from (A) are shown. (TIF) [file pone.0025336.s002.tif]
