# Supplementary material for: Regulation of Septin Dynamics by the Saccharomyces cerevisiae Lysine Acetyltransferase NuA4
Source: PLoS One. 2011 Oct 3;6(10):e25336. doi: 10.1371/journal.pone.0025336 (PMC3184947; doi:10.1371/journal.pone.0025336)
Supplement: Table S1 — Strains used in this study. (DOCX) [file pone.0025336.s003.docx]

**Table S1: Strain list**

| Strain | Genotype | Reference or Source |
| --- | --- | --- |
| YKB779 | *MATa ura3-52 lys2-801 ade2-101 trp1-∆200 leu2-∆1* | [43] |
| YKB780 | *MATα ura3-52 lys2-801 ade2-101 trp1-∆200 leu2-∆1* | [43] |
| YKB622 | *MATα can1∆::STE2pr-Sp_his5 lyp1∆ his3∆1 leu2∆0 ura3∆0 met15∆0 eaf1∆::NAT* | [9] |
| YKB995 | *MATα can1∆::STE2pr-Sp_his5 lyp1∆ his3∆1 leu2∆0 ura3∆0 met15∆0 eaf3∆::NAT* | [13] |
| YKB852 | *MATα can1∆::STE2pr-Sp_his5 lyp1∆ his3∆1 leu2∆0 ura3∆0 met15∆0 eaf5∆::NAT* | [9] |
| YKB623 | *MATα can1∆::STE2pr-Sp_his5 lyp1∆ his3∆1 leu2∆0 ura3∆0 met15∆0 eaf6∆::NAT* | Gift from N. Krogan |
| YKB853 | *MATα can1∆::STE2pr-Sp_his5 lyp1∆ his3∆1 leu2∆0 ura3∆0 met15∆0 eaf7∆::NAT* | [9] |
| YKB44 | *MATa ura3-52 lys2-801 ade2-101 trp1-∆200 leu2-∆1 eaf1∆::kanMX* | [13] |
| YKB1224 | *MATa ura3-52 lys2-801 ade2-101 trp1-∆200 leu2-∆1 eaf1∆::NAT* | This study |
| YKB1162 | *MATa ura3-52 lys2-801 ade2-101 trp1-∆200 leu2-∆1 eaf3∆::kanMX* | [13] |
| YKB658 | *MATa ura3-52 lys2-801 ade2-101 trp1-∆200 leu2-∆1 eaf5∆::TRP1* | [13] |
| YKB504 | *MATa ura3-52 lys2-801 ade2-101 trp1-∆200 leu2-∆1 eaf6∆::kanMX* | [13] |
| YKB530 | *MATa ura3-52 lys2-801 ade2-101 trp1-∆200 leu2-∆1 eaf7∆::kanMX* | [13] |
| YKB464 | *MATa ura3-52 lys2-801 ade2-101 trp1-∆200 leu2-∆1 yaf9∆::kanMX* | [13] |
| YKB494 | *MATa ura3-52 lys2-801 ade2-101 trp1-∆200 leu2-∆1 yng2∆::kanMX* | [13] |
| YKB1443 | *MATa his3∆1 leu2∆0 met15∆0 ura3∆0 CDC10-TAP::HIS3* | TAP Collection |
| YKB1455 | *MATa his3∆1 leu2∆0 met15∆0 ura3∆0 CDC11-TAP::HIS3* | TAP Collection |
| YKB1466 | *MATa his3∆1 leu2∆0 met15∆0 ura3∆0 SHS1-TAP::HIS3* | TAP Collection |
| YKB440 | *MATa his3∆1 leu2∆0 met15∆0 ura3∆0 ESA1-TAP::URA3* | [13] |
| YKB1807 | *MATa his3∆1 leu2∆0 met15∆0 ura3∆0 swe1∆::kanMX* | DMA collection |
| YKB1266 | *MATa swe1∆::kanMX eaf1∆::kanMX* | This study |
| YKB1648 | *MATa swe1::pGAL-SWE1-HA::LEU2* | Gift from A. Rudner |
| YKB1806 | *MATa swe1::pGAL-SWE1-HA::LEU2 eaf1∆::kanMX* | This study |
| YKB1312 | *MATα CDC11-GFP::HIS3* | This study |
| YKB1310 | *MATα CDC11-GFP::HIS3 eaf1∆::kanMX* | This study |
| YKB1385 | *MATα CDC11-GFP::HIS3 yng2∆::kanMX* | This study |
| YKB1376 | *MATα CDC11-GFP::HIS3 eaf3∆::kanMX* | This study |
| YKB1378 | *MATα CDC11-GFP::HIS3 eaf5∆::TRP1* | This study |
| YKB1379 | *MATα CDC11-GFP::HIS3 eaf6∆::kanMX* | This study |
| YKB1381 | *MATα CDC11-GFP::HIS3 eaf7∆::kanMX* | This study |
| YKB1383 | *MATα CDC11-GFP::HIS3 yaf9∆::kanMX* | This study |
| YKB1664 | *MATα CDC11-GFP::HIS3 gcn5∆::kanMX* | This study |
| YKB1668 | *MATα CDC11-GFP::HIS3 sas2∆::kanMX* | This study |
| YKB1672 | *MATα CDC11-GFP::HIS3 rtt109∆::kanMX* | This study |
| YKB1676 | *MATα CDC11-GFP::HIS3 elp3∆::kanMX* | This study |
| YKB1679 | *MATα CDC11-GFP::HIS3 hpa2∆::kanMX* | This study |
| YKB1682 | *MATα CDC11-GFP::HIS3 hat1∆::kanMX* | This study |
| YKB1796 | *MATα CDC11-GFP::HIS3 spt10∆::kanMX* | This study |
| YKB1800 | *MATα CDC11-GFP::HIS3 sas3∆::kanMX* | This study |
| YKB1878 | *MATα SHS1-GFP::HIS3* | This study |
| YKB2145 | *MATα CDC11-GFP::HIS3 eco1-203* | This study |
| YKB2138 | *MATa eco1-203 pep4∆ ::G418 ura3-52 leu2-3,112 his3-11,15 bar1 GAL+* | [58] |
| YKB2160 | *MATα CDC11-GFP::HIS3 hht1-hhf1::pWZ405-F2F9-LEU2 hht2-hhf2::pWZ403-F4F10-HIS3 [HHT2-HHF2::TRP1]* | This study; [55] |
| YKB2161 | *MATα CDC11-GFP::HIS3 hht1-hhf1::pWZ405-F2F9-LEU2 hht2-hhf2::pWZ403-F4F10-HIS3 [HHT2-hhf2∆4-19::TRP1]* | This study; [55] |
| YKB1804 | *MATα CDC11-GFP::HIS3 esa1Δ::HIS3 esa1(L254P)::URA3* | This study |
| YKB2254 | *MATa CDC11-GFP::HIS3 shs1∆TRP [pRS415::LEU2]* | This study |
| YKB2255 | *MATa CDC11-GFP::HIS3 shs1∆TRP [SHS1-HA_3_::LEU2]* | This study |
| YKB2518 | *MATα CDC11-GFP::HIS3 SHS1-HA_3_::kanMX* | This study |
| YKB2519 | *MATα CDC11-GFP::HIS3 shs1∆TRP [pRS415::LEU2]* | This study |
| YKB2520 | *MATα CDC11-GFP::HIS3 shs1∆TRP [SHS1::LEU2]* | This study |
| YKB2521 | *MATα CDC11-GFP::HIS3 shs1∆TRP [shs1-66∆::LEU2]* | This study |
| YKB2523 | *MATα CDC11-GFP::HIS3 shs1∆TRP [shs1-K16R-K19R-K57R-K82R-K204R-K443R-K478R-K488R-K536R::LEU2]* | This study |
| YKB2747 | *MATα CDC11-GFP::HIS3 shs1∆TRP [shs1-K488R-K492R-K500R-K505R-K509R-K515R-K535R-K536R::LEU2]* | This study |
| YKB2748 | *MATα CDC11-GFP::HIS3 shs1∆TRP [shs1-K478R-K488R-K492R-K500R-K505R-K509R-K515R-K535R-K536R::LEU2]* | This study |
| YKB2518 | *MATα CDC11-GFP::HIS3 shs1-66∆::NAT* | This study |
| YKB2519 | *MATα CDC11-GFP::HIS3 SHS1::NAT* | This study |
