# Supplementary material for: Regulation of Septin Dynamics by the Saccharomyces cerevisiae Lysine Acetyltransferase NuA4
Source: PLoS One. 2011 Oct 3;6(10):e25336. doi: 10.1371/journal.pone.0025336 (PMC3184947; doi:10.1371/journal.pone.0025336)
Supplement: Table S2 — Plasmids generated for this study. (DOCX) [file pone.0025336.s004.docx]

**Table S2: Plasmids generated for this study**

| **Plasmid** | **Name** | **Vector** | **Insert** | **Source** |
| --- | --- | --- | --- | --- |
| pKB65 | HA_3_ | pRS415 | *SHS1-HA_3_* | this study |
| pKB66 | WT | pRS415 | *SHS1* | this study |
| pKB67 | 66Δ | pRS415 | *shs1-66Δ* | this study |
| pKB68 | All-R | pRS415 | *shs1-K16-K19-K57-K88-K204-K443-K478-K488-K536R* | this study |
| pKB69 | 8R | pRS415 | *shs1-K488-K492-K500-K505-K509-K515-K535-K536R* | this study |
| pKB70 | 9R | pRS415 | *shs1-K478-K488-K492-K500-K505-K509-K515-K535-K536R* | this study |
